# Short-Term Effects of Air Pollution on Hospital Admissions of the Elderly with Respiratory and Cardiovascular Diseases Using a New Poisson-Generalized Lindley Model

**DOI:** 10.34172/jrhs.11533

**Published:** 2026-02-21

**Authors:** Soraya Moamer, Mostafa Leili, Javad Faradmal

**Affiliations:** ^1^Modeling of Noncommunicable Diseases Research Center, Institute of Health Sciences and Technology, Hamadan University of Medical Sciences, Hamadan, Iran; ^2^Department of Biostatistics, School of Public Health, Hamadan University of Medical Sciences, Hamadan, Iran; ^3^Department of Environmental Health Engineering, School of Public Health and Research Center for Health Sciences, Hamadan University of Medical Sciences, Hamadan, Iran

**Keywords:** Air pollution, Hospital admission, Respiratory disease, Cardiovascular disease, Overdispersion

## Abstract

**Background:**

Poisson and negative binomial (NB) regression models are commonly used to investigate the association between air pollution and hospital admissions for cardiovascular and respiratory diseases. This study utilized the new Poisson-generalized Lindley (NPGL) regression model to evaluate the relationship between air pollutants and daily hospital admissions for these diseases among elderly individuals.

**Study Design:**

An ecological cross-sectional study.

**Methods:**

The data related to daily air pollutant concentrations, meteorological parameters, and the number of hospitalizations for cardiovascular and respiratory patients were gathered from the Environmental Protection Organization, the General Directorate of Meteorology, Farshchian Heart Hospital, and Shahid Beheshti Hospital in Hamadan. Then, the relationship between air pollution and daily hospital admissions was assessed using Poisson, NB, and NPGL models.

**Results:**

The findings indicated that the accuracy of the regression coefficients estimated in the NPGL model was higher than that in the NB and Poisson models for most pollutants. Specifically, the relative risk for carbon monoxide (CO) was calculated at 1.307 (95% confidence interval: 1.270–1.345). Cardiovascular hospitalization increased by 30.7% for each unit increase in CO concentration. A significant and direct association was found between exposure to all pollutants, except for PM_2.5_, and hospitalization for respiratory diseases (*P*<0.05).

**Conclusion:**

Overall, there was a significant relationship between air pollution and hospital admissions for cardiovascular diseases (CVDs) and respiratory diseases among the elderly, particularly regarding CO. This study indicates the need for policymakers to implement health programs to mitigate the effects of air pollution on vulnerable elderly populations.

## Background

 Air pollution has been identified as one of the world’s major environmental problems with adverse effects on human health. It particularly affects the respiratory and circulatory systems.^1^ According to the World Health Organization, air pollution is responsible for seven million early deaths worldwide.^2^ It is known that growth in urbanization and industrialization is one of the factors that increases air pollution.^3^ The mortality rate increases with an increase in the concentration of air pollutants.^4^ Cardiovascular disease (CVD) is responsible for 31% of deaths worldwide, and air pollution leads to increased CVD complications and hospital admissions.^5^ Palpitations, heart rate variability, increased blood pressure, and arteriosclerosis are some of the cardiovascular complications associated with air pollution.^4^ In 2019, a study conducted in Yichang, China, reported that the association between fine particulate matter (PM_2.5_) and hospital admissions for CVDs and respiratory diseases was stronger in elderly people and during the cold season.^6^ The most deaths related to air pollution occur in low-income countries, such as Asian countries.^3^ The increase in the concentration of air pollutants is one of the most serious environmental challenges in Iran.^7^ This issue has placed Iran in seventh place in the global ranking of the most polluted countries. According to a 2018 meta-analysis performed in Iran, the increase in air pollutants, such as PM_2.5_, inhalable PM (PM_10_), nitrogen dioxide (NO_2_), sulfur dioxide (SO_2_), carbon monoxide (CO), and ozone (O_3_), leads to an increase in mortality and admissions to hospital due to CVDs and respiratory diseases.^3^ In addition, more than 8.6% of all deaths were attributed to PM_2.5 _pollution in 2015.^3^

 In a study conducted in Shiraz, vulnerable age groups, especially the elderly, were more sensitive to air pollution.^8^ For instance, CO, PM_10_, NO_2_, SO_2_, and O_3_ pollutants were found to have an adverse effect on the respiratory system of older people even at concentrations below the air quality standards. The association between air pollutants (exposure) and daily hospital admissions (outcome) is assessed by assuming that the number of hospital admissions follows a Poisson distribution.^9-11^ However, one of the properties of the Poisson distribution is that its mean and variance are equal. For real data cases, this property is often not present, implying that the variance of the data is greater than their mean. This is called overdispersion.^12^ In such a situation, an alternative solution to the problem of overdispersion is to use the negative binomial (NB) distribution. In 2017, Ardiles et al compared Poisson and NB regression models to examine the association between air pollution and hospitalizations for CVDs and respiratory diseases. They plotted the half-normal probability plot for both models. Based on this plot, the Poisson model demonstrated a lack of fit to the data, indicating that the Poisson model could not describe the data well.^13^ The other solution to the problem of overdispersion is to utilize the mixed Poisson distribution. In 2021, Altun introduced a mixed Poisson distribution, the generalized Poisson-Lindley distribution (NPGL), to solve the problem. In the NPGL distribution, the mean response is a random variable that follows the Lindley distribution, which was introduced by Ekhosuehi et al in 2018.^14^ In 2021, Altun expanded on this concept, demonstrating that the NPGL distribution can reduce to the geometric distribution and the Poisson-Lindley distribution for specific parameter values. This flexibility makes the NPGL distribution particularly effective for modeling data characterized by overdispersion, right skewness, and zero inflation. This makes it more flexible than the NB and Poisson distributions. Altun further showed how this distribution can be employed to model count data.^14^ To the best of our knowledge, the studies conducted so far in Hamedan to investigate the short-term health effects of pollution are all based on Poisson and NB models.^15-17^

 This study aims to fit and compare three Poisson, NB, and NPGL distributions to model the relationship between air pollutants and daily hospital admissions due to CVDs and respiratory diseases in Hamedan. This study seeks to investigate a vulnerable group of elderly people. In addition, the effects of meteorological parameters are controlled to examine the relationship between air pollution and hospitalization.

## Methods

###  Study design and setting

 This ecological and cross-sectional study was performed to determine the relationship between air pollution and hospital admissions for respiratory disease and CVDs among the elderly ( ≥ 65 years old) in the city of Hamedan, Iran, in 2018.

 Hamedan is one of the western provinces of Iran with an area of 20172 km^2^. This study was conducted in Hamedan (Geographical Coordinates: 34°48′ N, 31°48′ E), which is the capital city of Hamedan Province.^18^ According to a census performed in 2019, Hamedan had an estimated population of approximately 783,300 people.^19^ Hamedan is located at the foot of the Alvand Mountains at an altitude of 1,800 m above sea level. This region has cold winters and dry summers. Moreover, the average annual temperature and rainfall are 3°C and 339 mm, respectively.^20^

###  Daily hospital admission data

 In this study, all hospitalizations for respiratory diseases (ICD-10th revision: J00 to J99) and CVDs (ICD-10th revision: I00 to I99) in the elderly (65 years or older) were provided by Shahid Beheshti and Farshchian Cardiovascular Hospitals from 21 March 2018 to 20 March 2019, respectively. These hospitals are affiliated with Hamadan University of Medical Sciences. Data included age, date of admission, and diagnosis codes. Furthermore, pneumonia (J18-19), chronic obstructive pulmonary disease (COPD) (J40-47), and asthma (J45-46) were the causes of daily hospital admissions for respiratory diseases. Moreover, hospital admissions for cardiovascular outcomes according to the International Classification of Diseases included ischemic heart disease (IHD) (I20-25), myocardial infarction (MI) (I21-22), and stroke (I60-69). Since then, the average number of daily admissions for both diseases has been higher for older people than for people younger than 65 years. Additionally, the average daily admissions for both diseases were higher for people over 65 than for people under 65. This study was limited to older people.

###  Air pollutants and meteorological data

 Daily mean air pollutant data, including PM_10_, PM_2.5_, NO_2_, SO_2_, CO, and the maximum of 8-hour mean for O_3_, were obtained from the Department of Environment of Hamedan. In addition, meteorological data, including relative humidity (%), daily temperature (℃), and wind speed (km/h), which are possible confounding parameters^21^ for hospital admissions, were obtained from the Hamedan Meteorological Organization.

###  Preparation of the data

 Data preparation is considered one of the most important steps in modelling the relationship between air pollution and admissions to hospital. Therefore, the following steps were taken in this regard. First, missing data and outliers were separately checked for all three datasets, including hospitalizations, air pollutants, and meteorological variables. There were no missing values for hospitalization and meteorological data. However, for air pollutants, missing data were imputed using the multivariate method.^22^ Additionally, outlier values for pollutants were removed based on expert advice from the Environmental Protection Department. Second, the hospitalized cases were extracted according to age ≥ 65 years old. The number of hospital admissions was then counted daily. Finally, the information on daily hospital admissions was merged with the data related to air pollutants and meteorological parameters.

###  Variable selection

**Figure 1:**
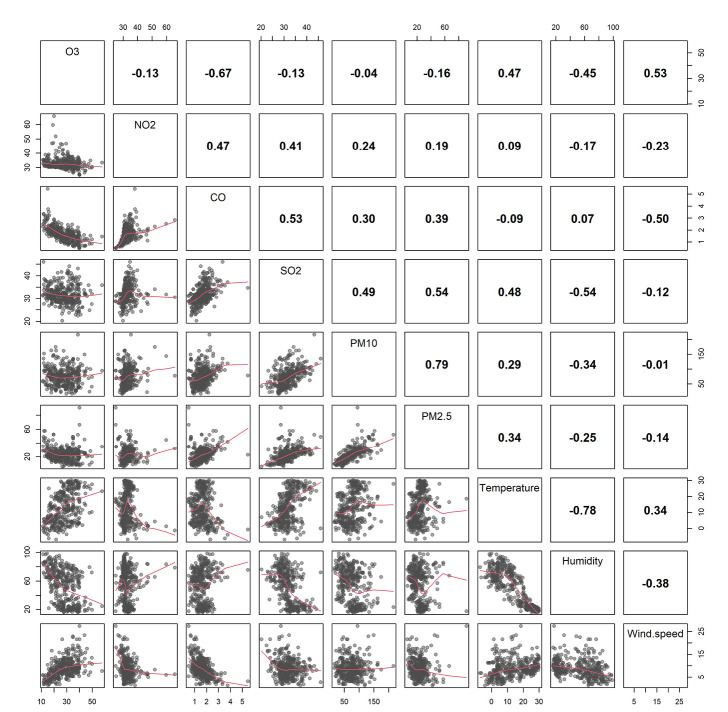
Spearman’s correlation coefficients (above the main diagonal) and the scatter plot (below the main diagonal) between different pollutants and meteorological variables

 In this part of the study, meteorological variables (humidity, temperature, and wind speed) and season were added to the model as potential confounders, and their effects were adjusted to determine the exact effects of pollutants on hospital admissions. Before fitting the models, the Spearman’s rank correlation coefficient was obtained between the pollutants and between the pollutants and the meteorological parameters, due to the non-normality of some variables (Figure 1).

 First, the Spearman correlation coefficient was used to examine the colinearity between the variables. There is a high and positive correlation between PM_10_ and PM_2.5_. Therefore, PM_2.5 _was chosen for two reasons. Initially, several studies in Iran reported that exposure to PM_2.5_, even at low concentrations, is associated with increased mortality and hospital admissions due to CVDs and respiratory diseases.^23,24^ Another reason for selecting PM_2.5 _was that there were fewer missing data for this pollutant than for PM_10_. Due to the high and negative correlation between temperature and relative humidity, only temperature was included in the regression model. The decision to use the temperature variable was based on the fact that several studies have shown that there is a statistically significant correlation between air pollution and hospital admissions in the warm and cold seasons.^25,26^

###  Model fitting

 In the present study, Poisson and NB generalized linear models, as well as the NPGL regression model, were employed to assess the short-term effects of air pollution on hospitalizations for CVDs and respiratory diseases in Hamedan. A logarithmic function was utilized for the modelling,^14^ while adjusting for other variables (i.e., temperature, wind speed, and season). For all three regression models, the regression coefficients were also estimated using the maximum likelihood method. Regression coefficients represent the log-relative rate of daily hospital admissions associated with a unit increase in air pollutants. Total respiratory disease, total CVD, chronic obstructive pulmonary disease (COPD), asthma, pneumonia, IHD, myocardial infarction (MI), and stroke were analyzed separately.

###  Comparison of the models

 The standard error (SE) of coefficients was used to compare the models. Additionally, to compare the associations across models, the results were presented as relative risk (RR) and 95% confidence intervals (CIs) for every one-unit increase in the respective pollutant variable. The Akaike information criterion (AIC) was also utilized as a key metric for model selection, allowing for the assessment of model fit. Lower AIC values indicate a better fit, and this criterion was instrumental in determining the most appropriate model for analyzing the associations between air pollution and hospital admissions for respiratory diseases.

###  Software

 All analyses were conducted using R software (version 4.2.1) for data preparation and fitting the models.^27^ The dplyr package to count daily hospitalizations and the mtsdi package were employed to impute missing data.^28,29^ In addition, the glm and glm.nb functions were applied to fit Poisson and NB regression models, respectively. In the NPGL regression model, to estimate the regression coefficients, all estimating equations were formed based on the maximum likelihood estimation method. The maximum likelihood method was used to estimate the parameters for the NPGL regression model. The log-likelihood function specific to the NPGL model was derived and then maximized to obtain the parameters, including regression coefficients and an additional parameter. Given the non-linear nature of these relationships, the Newton-Raphson method was applied, starting with initial parameter values. Moreover, the log-likelihood function was approximated using a second-degree Taylor expansion, requiring the calculation of the gradient vector and the Hessian matrix. Parameter values were iteratively updated until convergence was achieved, with initial estimates typically derived from the NB model, all implemented in R software.

## Results

###  Descriptive analysis

 Overall, 59,687 patients were admitted to Shahid Beheshti and Farshchian CVD Hospitals from March 21, 2018, to March 20, 2019. The total of hospital admissions due to CVD (n = 47,214, 79%) was higher than the total of hospital admissions due to respiratory diseases (n = 12,473, 21%). The association between air pollution and hospital admissions was analyzed for 32,798 people who were older than 65 years during the study period.

 The average daily temperature in Hamedan in 2018, the relative humidity, and the average wind speed were 12.91℃, 52.86%, and 8.86 km/h, respectively, indicating the cold weather in Hamedan. Among the pollutants, PM_10_ with an average of 74.56 ± 30.70 μg/m^3^ and PM_2.5 _with an average of 23.61 ± 9.98 μg/m^3^ had the highest and lowest mean concentrations, respectively (Table 1).

**Table 1:** Summary Statistics for the daily concentration of air pollution and meteorological variables in Hamedan from 21st March 2018 to 20th March 2019

**Variables**	**Concentration/Day**						
	**Mean**	**SD**	**Min.**	**P25**	**P50**	**P75**	**Max.**
Air pollutants (24-hour average)							
PM_10_ (μg/m^3^)	74.56	30.70	16.21	50.00	75.20	92.29	216.21
PM_2.5_ (μg/m^3^)	23.61	9.98	5.89	17.08	23.43	28.04	90.97
NO_2_ (μg/m^3^)	32.91	4.10	24.91	30.68	31.83	34.18	65.78
SO_2_ (μg/m^3^)	31.80	3.99	20.25	29.13	31.62	33.93	46.01
CO (mg/m^3^)	1.65	0.59	0.49	1.26	1.61	1.96	5.45
O_3_ (8 h average) (μg/m^3^)	27.88	8.44	11.27	21.32	27.54	34.22	57.68
Weather variables							
Temperature (^°^C)	12.91	9.66	7.10	4.10	12.20	21.60	30.10
Relative humidity (%)	52.86	24.03	16.00	26.75	57.50	72.75	98.62
Wind speed (km/h)	8.86	4.13	1.35	6.15	8.10	11.25	27.45

*Note*. SD: Standard deviation; P25: 25th percentile; P50: 50th percentile (median); P75: 75th percentile; Min.: Minimum; Max.: Maximum; O_3_: Ozone; NO_2_: Nitrogen dioxide; CO: Carbon monoxide; SO_2_: Sulfur dioxide; PM_2.5_: Fine particulate matter; PM_10_: Inhalable particulate matter

 For elderly people and in the subgroup of CVD, IHD was the most common cause of hospital admission, with an average of 25 cases per day, while COPD had the highest number of hospital admissions among respiratory diseases, with an average of more than 2 cases per day (Table 2).

**Table 2:** Summary statistics for daily hospital admissions in Hamedan from 21st March 2018 to 20th March 2019

**Variables**	**ICD-10 Code**	**Number of admissions/Days**				
		**Sum**	**Mean**	**SD**	**Minimum**	**Maximum**
Total cardiovascular diseases	I00-I99	47214	129.00	53.27	21.00	264.00
Adults (15–64)		21715	59.05	30.13	4.00	147.00
Elderly ( ≥ 65)		25499	69.86	30.83	7.00	156.00
Sub-diagnoses for elderly						
Ischemic heart disease	I20-I25	9098	25.06	13.14	2.00	71.00
Myocardial infarction	I21-I22	1220	4.25	2.60	1.00	17.00
Stroke	I60-I69	94	1.84	1.04	1.00	7.00
Total respiratory disease	J00-J99	12473				
Age (year)						
Adults (15–64)		5174	2.70	2.01	1.00	20.00
Elderly ( ≥ 65)		7299	3.44	2.20	1.00	16.00
Sub-diagnoses for elderly						
Pneumonia	J18-J19	243	1.44	0.69	1.00	4.00
Chronic obstructive pulmonary disease	J40-J47	668	2.37	1.56	1.00	10.00
Asthma	J45-J46	78	1.16	0.44	1.00	3.00

*Note*. ICD-10: International Classification of Diseases, tenth revision; SD: Standard deviation.

 The correlation analysis revealed that the SO_2_, NO_2_, and CO positively and moderately correlated with each other. PM_10_ and PM_2.5 _displayed the stronger correlation (r = 0.76, *P*< 0.05). The correlation of temperature with PM_10_, PM_2.5_, O_3_, NO_2_, and SO_2_ was positive, while the correlation of humidity and wind speed with PM_10_, PM_2.5_, O_3_, NO_2_, and SO_2_ was negative (Figure 1).

###  Model results of hospital admissions for cardiovascular disease

 The results of fitting the regression models for CVDs and respiratory diseases are presented in Tables 3 and 4, respectively. A significant association was found between air pollution and admission to hospital for all CVDs (I00-I99) in all three models (*P*< 0.05). For the majority of pollutants, the precision of the regression coefficients estimated in the NPGL model was higher, while the SE was lower in this model than in the NB and Poisson models. Based on the NPGL model, the relative risk for CO was 1.307 (95% CI: 1.270-1.345). For every one-unit increase in CO concentration, hospitalizations due to CVD increased by 30.7%. Furthermore, the AIC values indicated that the Poisson model generally offers a better fit for the data compared to the NB and NPGL models for most CVD results (Table 3).

**Table 3:** The results of regression model fitting: the effect of pollutants on elderly admissions due to various cardiovascular diseases

**Hospital admissions**	**Poisson**		**Negative binomial**		**Poisson-generalized Lindley**	
	**β (SE)**	**Relative risk (95% CI)**	**β (SE)**	**Relative risk (95% CI)**	**β (SE)**	**Relative risk (95% CI)**
Total cardiovascular						
O_3_	0.011 (0.001)	1.011 (1.008, 1.013)	0.012 (0.005)	1.012 (1.002, 1.021)	0.012 (0.001)	1.012 (1.010, 1.014)
NO_2_	0.015 (0.002)	1.016 (1.012, 1.019)	0.016 (0.007)	1.016 (1.003, 1.029)	0.016 (0.001)	1.016 (1.014,1.017)
CO	0.225 (0.017)	1.252 (1.210, 1.295)	0.268 (0.070)	1.307 (1.139, 1.500)	0.267 (0.015)	1.307 (1.270, 1.345)
SO_2_	-0.009 (0.002)	0.991 (0.986, 0.996)	-0.010 (0.009)	0.990 (0.972, 1.008)	-0.012 (0.001)	0.988 (0.987, 0.990)
PM_2.5_	0.00 (0.001)	1.000 (0.999, 1.002)	0.00 (0.003)	1.000 (0.995, 1.006)	-0.0003 (0.001)	1.000 (0.998, 1.002)
AIC	3011.470		2956.322		2776.501	
Stroke						
O_3_	-0.013 (0.023)	0.987 (0.943, 1.033)	0.010 (0.036)	1.010 (0.941, 1.083)	-0.006 (0.004)	0.994 (0.986, 1.002)
NO_2_	-0.019 (0.032)	0.981 (0.921, 1.045)	-0.026 (0.047)	0.975 (0.889, 1.069)	-0.009 (0.004)	0.991 (0.984, 0.998)
CO	-0.174 (0.325)	0.840 (0.436, 1.620)	-0.258 (0.507)	0.772 (0.286, 2.087)	-0.242 (0.064)	0.785 (0.692, 0.891)
SO_2_	0.056 (0.040)	1.058 (0.978, 1.144)	0.084 (0.064)	1.088 (0.960, 1.233)	0.069 (0.004)	1.072 (1.064, 1.079)
PM_2.5_	-0.018 (0.004)	0.982 (0.974, 0.990)	-0.010 (0.021)	0.990 (0.949, 1.032)	- 0.013 (0.005)	0.987 (0.978, 0.996)
AIC	3188.043		2997.122		2821.254	
Myocardial infarction						
O_3_	0.003 (0.006)	1.003 (0.991, 1.015)	0.003 (0.006)	1.002 (0.991, 1.015)	0.002 (0.002)	1.002 (0.999, 1.006)
NO_2_	0.015 (0.007)	1.015 (1.001, 1.029)	0.015 (0.007)	1.015 (1.001, 1.029)	0.015 (0.002)	1.015 (1.012, 1.018)
CO	0.055 (0.085)	0.090 (0.894, 1.247)	0.055 (0.085)	1.058 (0.894, 1.247)	0.056 (0.029)	1.058 (1.000, 1.119)
SO_2_	0.011 (0.001)	0.011 (0.990, 1.033)	0.011 (0.011)	1.013 (0.990, 1.033)	0.013 (0.002)	1.013 (1.010, 1.017)
PM_2.5_	0.001 (0.003)	0.001 (0.995, 1.008)	0.001 (0.003)	1.001 (0.995, 1.008)	0.001 (0.002)	1.001 (0.997, 1.005)
AIC	3097.153		3014.030		2987.761	
Ischemic heart disease						
O_3_	0.005 (0.002)	1.005 (1.000, 1.009)	0.005 (0.002)	1.006 (0.995, 1.018)	0.007 (0.001)	1.007 (1.005, 1.009)
NO_2_	0.010 (0.003)	1.010 (1.005, 1.015)	0.010 (0.003)	1.010 (0.995, 1.026)	0.010 (0.001)	1.010 (1.008, 1.012)
CO	0.227 (0.028)	1.255 (1.187, 1.327)	0.227 (0.028)	1.330 (1.126, 1.573)	0.285 (0.018)	1.329 (1.283, 1.378)
SO_2_	-0.012 (0.004)	0.988 (0.981, 0.996)	-0.012 (0.004)	0.989 (0.967, 1.010)	-0.012 (0.001)	0.989 (0.986, 0.990)
PM_2.5_	0.00 (0.001)	1.000 (0.998, 1.003)	0.000 (0.001)	1.001 (0.994, 1.006)	-0.001 (0.001)	0.999 (0.997, 1.002)
AIC	3243.664		2981.201		2877.065	

*Note*. SE: Standard error; CI: Confidence interval; O_3_: Ozone; NO_2_: Nitrogen dioxide; CO: Carbon monoxide; SO_2_: Sulfur dioxide; PM_2.5_: Fine particulate matter; AIC: Akaike information criterion.

**Table 4:** The results of regression model fitting: the effect of pollutants on elderly admissions due to various respiratory diseases

**Hospital admissions**	**Poisson**		**Negative Binomial**		**Poisson-Generalized Lindley**	
	**β (SE)**	**Relative Risk (95% CI)**	**β (SE)**	**Relative Risk (95% CI)**	**β (SE)**	**Relative Risk (95% CI)**
Total respiratory						
O_3_	0.015 (0.006)	1.015 (1.002, 1.027)	0.016 (0.008)	1.016 (1.001, 1.032)	0.017 (0.002)	1.017 (1.013, 1.020)
NO_2_	-0.009 (0.008)	0.991 (0.976, 1.007)	-0.009 (0.010)	0.991 (0.971, 1.012)	-0.008 (0.002)	0.992 (0.988, 0.995)
CO	0.310 (0.012)	1.364 (1.173, 1.584)	0.343 (0.108)	1.409 (1.140, 1.741)	0.352 (0.030)	1.422 (1.342, 1.507)
SO_2_	0.022 (0.012)	1.023 (1.00, 1.046)	0.024 (0.015)	1.024 (0.995, 1.055)	0.025 (0.002)	1.026 (1.022, 1.029)
PM_2.5_	-0.011 (0.004)	0.989 (0.982, 0.997)	-0.011 (0.005)	0.989 (0.980, 0.998)	-0.011 (0.002)	0.989 (0.985, 0.993)
AIC	2625.705		2422.612		2311.403	
Pneumonia						
O_3_	0.012 (0.013)	1.012 (0.986, 1.039)	0.016 (0.008)	1.016 (1.001, 1.032)	0.015 (0.003)	1.015 (1.009, 1.021)
NO_2_	-0.01 (0.017)	0.990 (0.958, 1.024)	-0.009 (0.10)	0.991 (0.971, 1.012)	-0.008 (0.002)	0.992 (0.987, 0.997)
CO	0.078 (0.172)	1.081 (0.771, 1.514)	0.343 (0.108)	1.409 (1.140, 1.741)	0.054 (0.044)	1.055 (0.967, 1.151)
SO_2_	0.073 (0.023)	1.076 (1.028, 1.127)	0.024 (0.015)	1.024 (0.995, 1.055)	0.083 (0.003)	1.086 (1.081, 1.091)
PM_2.5_	-0.010 (0.009)	0.990 (0.973, 1.007)	-0.011 (0.005)	0.989 (0.980, 0.998)	-0.009 (0.003)	0.991 (0.985, 0.997)
AIC	2698.852		2502.411		2409.575	
Asthma						
O_3_	-0.018 (0.025)	0.982 (0.935, 1.032)	-0.019 (0.020)	0.981 (0.932, 1.033)	-0.019 (0.005)	0.982 (0.973, 0.990)
NO_2_	0.009 (0.031)	1.009 (0.949, 1.072)	0.008 (0.032)	1.008 (0.946, 1.074)	0.010 (0.004)	1.010 (1.003, 1.018)
CO	0.288 (0.306)	1.334 (0.732, 2.432)	0.301 (0.322)	1.352 (0.719, 0.254)	0.273 (0.070)	1.314 (1.145, 1.507)
SO_2_	-0.032 (0.046)	0.968 (0.884, 1.060)	-0.035 (0.048)	0.966 (0.879, 1.061)	-0.031 (0.004)	0.969 (0.962, 0.977)
PM_2.5_	-0.026 (0.016)	0.974 (0.944, 1.005)	-0.026 (0.016)	0.974 (0.943, 1.006)	-0.025 (0.005)	0.975 (0.965, 0.985)
AIC	2749.505		2673.204		2551.103	
Chronic obstructive pulmonary disease						
O_3_	0.020 (0.008)	1.020 (1.005, 1.036)	0.022 (0.009)	1.022 (1.003,1.040)	0.023 (0.002)	1.023 (1.019, 1.027)
NO_2_	0.001 (0.010)	1.001 (0.982, 1.021)	0.001 (0.012)	1.001 (0.978, 1.024)	0.001 (0.002)	1.001 (0.997, 1.004)
CO	0.407 (0.092)	1.502 (1.254, 1.798)	0.448 (0.119)	1.565 (1.240, 1.976)	0.477 (0.034)	1.612 (1.509, 1.721)
SO_2_	0.030 (0.015)	1.003 (0.974, 1.033)	0.004 (0.117)	1.004 (0.970, 1.039)	0.006 (0.002)	1.006 (1.002, 1.010)
PM_2.5_	-0.010 (0.005)	0.990 (0.981, 1.000)	-0.010 (0.005)	0.990 (0.979, 1.00)	-0.011 (0.002)	0.989 (0.984, 0.994)
AIC	2601.693		2518.610		2378.901	

*Note*. SE: Standard error; CI: Confidence interval; O_3_: Ozone; NO_2_: Nitrogen dioxide; CO: Carbon monoxide; SO_2_: Sulfur dioxide; PM_2.5_: Fine particulate matter; AIC: Akaike information criterion.

 In the stroke subgroup, the NPGL and NB models showed a significant association between CO and admission to the hospital. In the NPGL model, the SE of the estimated regression coefficients for this pollutant was 44% lower than that in the NB model. Regarding the stroke analysis, the AIC values demonstrated that the NPGL model provides the best fit, with an AIC of 2821.25, followed by the NB model with an AIC of 2997.12, and finally the Poisson model with an AIC of 3188.04. In all three models, there was a significant relationship between NO_2_ exposure and hospitalization for MI. In the NPGL model, the estimated RR for NO_2_ was more precise than that in the NB and Poisson models (RR = 1.307, 95% CI: 1.270-1.345, Table 3).

###  Model results of hospital admissions for respiratory diseases

 Based on the results of the fitted models for the association between air pollution and hospitalization for respiratory diseases, the SE values of the fitted coefficients for all pollutants in the NPGL model were lower compared to the NB and Poisson models. This finding was observed in different subgroups of respiratory diseases. It should be mentioned that all results were obtained by adjusting for the effects of disturbance factors (temperature, wind speed, and season). For total respiratory admissions, the AIC values confirmed that NPGL was the most appropriate model, with an AIC of 2311.40, followed by the NB model with an AIC of 2422.61. This suggests that the NPGL model provides a better fit for the data (Table 4).

 A significant and positive association was observed between exposure to all pollutants except PM_2.5 _and admission to hospital for all respiratory diseases based on the RR estimated in the NPGL model (*P* < 0.05). CO had the highest RR among the pollutants (RR = 1.422, 95% CI: 1.342–1.507).

 In the pneumonia subgroup, all pollutants, except CO, displayed a considerable association with hospitalizations in the NPGL model (*P* < 0.05). Regarding asthma, there was a significant association between exposure to pollutants and the risk of admission to hospital for asthma (*P* < 0.05). This correlation was found to be positive for NO_2_ and CO but negative for O_3_, SO_2_, and PM_2.5_. The results further demonstrated that for every one-unit increase in O_3_ pollutant concentration, the risk of hospitalization for COPD increased by 2.3% (RR = 1.023, 95% CI: 1.019–1.027, Table 4).

## Discussion

 Air pollution is a mixture of gaseous substances and PM that decreases life expectancy. This serious environmental challenge can impose considerable direct and indirect costs on society.^30^ In 2019, a review study examined the harmful effects of short-term and long-term exposure on the cardiovascular system. It was revealed that the burden of deaths from CVD was greater than previously imagined.^31^ Several studies suggested that air pollution not only contributes to cancer and chronic lung disease but also has adverse effects on the cardiovascular system. Moreover, air pollutants penetrate the lower respiratory tract and can help the absorption of organic substances, heavy metals, and other toxic substances, leading to the production of free radicals in the lungs and blood.^32,33^ As previously mentioned, this study investigated the association between air pollution and hospitalization of elderly people due to CVDs and respiratory diseases in Hamedan from March 1, 2018, to March 21, 2019.

 Our findings confirmed a positive and significant association between NO_2_ and hospital admissions for CVD (I00-I99). This association was also consistent with the subgroup of IHD and MI. This finding is in line with the results of other research.^34^ Short-term exposure to NO_2_ leads to cardiac dysfunction due to physiological mechanisms, such as inflammatory responses and changes in autonomic nervous control of the heart.^35^ However, some toxicological studies have reported that NO_2_ does not directly cause respiratory diseases. Nonetheless, this pollutant prepares the ground for various bacterial and viral infections of the respiratory system.^36^ A study conducted in Hamadan assessed the short-term effects of air pollution on respiratory hospitalizations. The analysis revealed a direct and significant association between NO₂ levels and an increase in hospital admissions for respiratory diseases.^21^ The influence of temperature as a confounding factor was controlled in the present study. Another study in Ahvaz showed that for every 1 μg/m^3^ increase in NO_2_ concentration in lag0, the risk of hospitalization increased by 0.06%.^37^ Conversely, in the present study, the risk of cardiovascular hospital admissions increased by 1.6%. The difference in these values can be explained by the differences in study year, data analysis method, and sample size. In line with the findings of this research, a positive association was observed between NO_2_ and hospitalization for IHD in a study performed in Hanoi, Vietnam.^38^

 Regarding the impact of NO_2_ on hospitalization of patients with respiratory diseases, a positive and significant association was found in the only asthma subgroup. Asthma is a chronic inflammatory lung disease, and people with asthma are sensitive to inhaled irritants (e.g., many air pollutants). Çapraz et al reported a significant association between NO_2 _and asthma. Therefore, the number of asthma hospitalizations increased by 2.3% per 10 units of NO_2_.^10^

 One of the most important sources of O_3_ is the combustion of fossil fuels.^39^ According to the findings of this study, the increase in O_3_ concentration led to an increase in the hospitalization of patients with CVD (I00-I99). Furthermore, this relationship was observed in the MI and IHD subgroups. This finding is consistent with the results of several other studies.^40,41^ A cohort study of 2.7 million people using the Cox proportional hazard model demonstrated a positive and significant association between long-term exposure to O_3_ and hospitalization for MI and stroke (HR = 1.062, 95% CI: 1.041–1.084). This relationship was stronger among older age groups (i.e., 65 years and older).^42^ On the other hand, Jin et al, in a cohort study on elderly people, concluded that long-term exposure to O_3_ increases the incidence of stroke (HR = 1.0026, 95% CI: 1.0023–1.0028),^43^ which contradicts our results, indicating that there was no significant relationship regarding stroke. This inconsistency may be due to the different concentrations of air pollution, people’s behavior, the different designs of the studies, environmental conditions, and social and economic status in various regions.

 The results of this study revealed a positive and significant relationship between O_3_ and admission to hospital due to total respiratory diseases and asthma, which conforms to the findings of some studies.^44,45^ Short-term exposure to O_3_ causes changes in chemical indices and lung structure. This pollutant enters the respiratory tract and causes pneumonia.^35^ Based on the NPGL model, exposure to O_3_ leads to an increase in hospital admissions for pneumonia. In contrast to this statement, 9-year data from Bangkok, Thailand, showed an inverse and significant association between the daily admissions of patients with pneumonia and air pollution.^36^ This inconsistency can be explained by the difference in weather conditions and the concentration of O_3_. This is because the average annual temperature and the average concentration of O_3_ are higher in Bangkok compared to Hamedan.

 In this investigation, a significant and inverse correlation for the PM_2.5 _was only found for the subgroup of stroke patients, which contradicts the findings of other studies.^46,47^ This finding may be attributed to the harvesting effect, which implies that highly sensitive individuals may die shortly after exposure to pollution and before being hospitalized, leading to an apparent decrease in hospitalization rates in the following days. Additionally, it could be due to unadjusted confounding factors (e.g., behavioral influences on days with very high pollution). Furthermore, this factor may be related to the low number of stroke cases in the stroke subgroup. PM is released by volcanoes, dust storms, grassland fires, tobacco burning, diesel engines, power plants, road dust, and industrial processes.^39^ Research indicates that PM_2.5 _has harmful effects, especially on the cardiovascular system.^32^ PM causes numerous pathophysiological changes (e.g., inflammation and oxidative stress) in various parts of the cardiovascular system, eventually resulting in the occurrence of CVD.^48^ In 2018, a systematic review based on 22 studies performed between 2010 and 2016 aimed to provide evidence on the impact of air pollution on the risk of hospitalization. The results demonstrated that PM_2.5_ has a stronger impact on hospital admissions for CVDs and respiratory diseases compared to other pollutants.^47^ A study in the United States found that the risk of hospitalization for heart failure on the same day of exposure increased by 1.28% when PM_2.5_ concentrations increased by 10 units.^46^ The reason for the significance of PM_2.5_ could be the high sample size in their study, so that their analysis was limited to the 204 US counties with more than 200,000 inhabitants. Similarly, a time-series study of 75 cities in the US showed that for every 10-unit increase in PM_2.5 _exposure, deaths from heart attack and stroke increased by 1.22% and 1.76%, respectively.^49^ A multicenter case-crossover study in Vietnam with 10 million participants confirmed that increases in air pollution were associated with increases in hospital admissions for CVD. For example, a positive and significant correlation was found for PM_2.5 _in the province of Hanoi, the capital of Vietnam. The effects of pollutants on hospital admissions of people over 65 years of age were greater than those of individuals under 65 years of age.^50^ In contrast to the present study, the significance of PM_2.5 _may be due to the design of their study (a case-crossover study). Another study in Europe indicated that the risk of stroke increased by 40% for every 5 µg per m^3^ increase in PM_2.5_ exposure.^51^ Based on their study, data from 11 cohorts were collected, and the occurrence of a first stroke was evaluated. Random-effects meta-analysis was used for pooled effect estimation. The difference in the method of data analysis was the reason for the contradictory findings of their study and the present study.

 COPD, asthma, and acute lower respiratory tract infections are the leading causes of serious illness and death in the world.^52^ Our findings revealed an inverse association between these diseases and PM_2.5_, which is not consistent with the results of other studies. A study in China was designed to estimate the short-term effects of PM exposure on hospitalizations for pneumonia, COPD, and asthma. For COPD, these two pollutants were significantly associated with hospitalization. Moreover, the short-term effects of PM_2.5_ were significant at a delay of 6–16 days, and the effects of PM_10_ were noticeable at a delay of 7–11 days. In addition, the effects of air pollutants were more pronounced in the warm season than in the cold season, and women and individuals over 65 years had a higher risk of PM_2.5 _exposure. Conversely, these estimates were not statistically significant for pneumonia admissions. However, there was a positive association for both PM_2.5 _and PM_10 _for admissions to hospital due to COPD.^53^

 Based on the findings of this study, there was a direct and significant association between SO_2_ and hospitalization for MI and stroke. In this regard, Nhung et al concluded that SO_2_ has a positive association with daily hospital admissions for stroke. Thus, for each interquartile range increase in SO_2_ concentration, the daily stroke rate increased by 6.7%.^38^ They further indicated that SO_2_ is usually released during the combustion of fossil fuels (e.g., in domestic heating systems and motor vehicles). It is one of the principal pollutants in many parts of the world. Short-term exposure to SO_2_ causes plasma fibrinogen, oxidative stress, and inflammatory factors, leading to an increase in blood viscosity and thus CVD.^54^ Furthermore, the function of the respiratory system decreases due to the constriction of the bronchial tubes.^36^ Moreover, exposure to SO_2_ is associated with an increase in hospitalization due to COPD and pneumonia. However, there is an inverse relationship with asthma.

 Based on the NPGL model, a strong and positive correlation was found between CO exposure and admissions to hospital for CVDs and respiratory diseases in the present study, which corroborates the findings of other studies.^13,55^ Disturbances in the release of oxygen in the tissues due to the binding of CO to hemoglobin impair the function of the heart and cause cardiovascular events, such as MI.^3^ In addition, toxicological findings demonstrate that exposure to CO can have direct effects on the respiratory system.^36^ Rozita et al observed that exposure to CO increases the risk of admissions to hospital due to CVDs and respiratory diseases.^1^ Additionally, the study identified women, children, and the elderly as groups at high risk from air pollution. Another study in Shiraz reported that a 1 µg/m^3^ increase in CO levels increased the risk of hospitalization for IHD by 52% on the day of exposure.^8^

 In the present study, an NPGL regression model was first used in Hamedan to assess the association between air pollution and hospitalization of cardiovascular and respiratory patients. On the other hand, the effects of meteorological parameters (e.g., temperature, humidity, and wind speed) were controlled as confounding variables. However, most studies ignore these parameters. One of the limitations of the study was the missing values for a number of pollutants. This was an observational study; therefore, many confounding factors (e.g., socioeconomic status, smoking status, underlying disease, and the like) could influence the association between air pollution and patient admissions to the hospital. Accordingly, it is suggested that future studies control the effect of these variables. On the other hand, a case-crossover design can be used to control confounding factors. Another limitation of the present study was that the data only covered a one-year period, which may not capture long-term patterns or unusual events. Thus, it is suggested that future research benefit from using multi-year data.

**Highlights**

- Air pollution raised cardiovascular/respiratory hospital admissions of the elderly.
- A new statistical model (New Poisson-generalized Lindley) proved to be more accurate than traditional Poisson and negative binomial (NB) models for this analysis.
- Carbon monoxide had the strongest effect, increasing cardiovascular admissions and respiratory admissions by 30.7% and 42.2% per unit increase, respectively.
- Policymakers must protect the elderly from the health effects of air pollution.

## Conclusion

 In this study, the estimates obtained from the NPGL regression model were more accurate compared to the NB and Poisson models. Based on the results of the NPGL model, there was a significant association between air pollution and hospitalizations of cardiovascular and respiratory patients. This association was stronger for the CO compared to other pollutants. On the other hand, our study was limited to the sensitive group of older people. It is, therefore, necessary for policymakers to plan and implement health schemes in order to reduce the impact of air pollution on elderly people.

## Acknowledgments

 The authors are grateful to the Department of Environment of Hamadan, Hamadan University of Medical Sciences, and Hamedan Meteorological Organization for providing air pollutant concentration data, admission data (Shahid Beheshti Hospital and Farshchian Cardiovascular Hospital), and climate data, respectively. Moreover, we thank the Vice-Chancellor of Research and Technology of Hamadan University of Medical Sciences. This article is part of a Ph.D. thesis (No. 14020205686) in biostatistics submitted to Hamadan University of Medical Sciences. Finally, the authors would like to appreciate the Clinical Research Development Unit of Shahid Beheshti Hospital, Hamadan University of Medical Sciences, Hamadan, Iran, for their assistance in writing the present manuscript.

## Competing Interests

 The authors declare they have no conflict of interests.

## Data Availability Statement

 Due to restrictions related to our internal review board policy, the dataset is only available upon reasonable request from the corresponding author.

## Ethical Approval

 The Ethics Committee of Hamadan University of Medical Sciences approved the study protocol (ethical code IR.UMSHA.REC.1400.410). In addition, individuals’ information (e.g., first and last names) was published collectively and in the form of index reports.

## Funding

 This research received no specific grant from funding agencies in the public, commercial, or not-for-profit sectors.

 Intelligence Use Disclosure

 We hereby declare the following regarding the use of artificial intelligence (AI) tools in our manuscript: (a) AI tools were used “only for language editing and grammar improvement” in the preparation of this manuscript; (b) No AI tools were used for data analysis, interpretation, or generation of any scientific content; (C) The final content, scientific integrity, and originality of the work are entirely the responsibility of the authors.
